# Extreme Drug Resistance in *Acinetobacter baumannii* Infections in Intensive Care Units, South Korea

**DOI:** 10.3201/eid1508.080772

**Published:** 2009-08

**Authors:** Young Kyoung Park, Kyong Ran Peck, Hae Suk Cheong, Doo-Ryeon Chung, Jae-Hoon Song, Kwan Soo Ko

**Affiliations:** Sungkyunkwan University School of Medicine, Suwon, South Korea (Y.K. Park, K.R. Peck, H.S. Cheong, D.-R. Chung, J.-H. Song, K.S. Ko); Asian-Pacific Research Foundation for Infectious Diseases, Seoul, South Korea (J.-H. Song, K.S. Ko); 1 These authors contributed equally to this article.

**Keywords:** Antimicrobial resistance, extremely drug resistant, intensive care units, Acinetobacter baumannii infections, South Korea, bacteria, letter

**To the Editor:** *Acinetobacter* spp. have emerged as a cause of nosocomial infections, especially in intensive care units (ICUs). In South Korea, *Acinetobacter* spp. was ranked as the third most frequently found pathogen in ICUs (*1*). With the emergence of multidrug-resistant (MDR) or pandrug-resistant (PDR) isolates, few drugs are now available to treat MDR or PDR *Acinetobacter* infections; polymyxins are the only therapeutic option in many cases (*2*). Current polymyxin resistance rates among *Acinetobacter* isolates are low worldwide (*3*). We report the emergence of extreme drug resistance (XDR) in *A. baumannii* isolates from patients in ICUs of Samsung Medical Center in Seoul, South Korea. These isolates were resistant to all tested antimicrobial drugs, including polymyxin B and colistin, to which PDR isolates are normally susceptible.

Sixty-three nonduplicate *Acinetobacter* spp. isolates were collected from the ICUs from April through November 2007. Species identification was performed based on partial RNA polymerase β-subunit gene sequences, amplified rDNA restriction analysis, and the gyrase B gene–based multiplex PCR method (*3*). Forty-four isolates were identified as *A. baumannii*: 9 as genomic species 3, six as genomic species 13TU, 2 as *A. baumannii*-like species, and 1 each as *A. junnii* and genomic species 10.

In vitro susceptibility testing was performed and interpreted by using the broth microdilution method according to the Clinical and Laboratory Standards Institute guidelines (*4*). Colistin and polymyxin B resistances were defined as MIC >4 mg/L (*4*). MDR was defined as characterized by resistance to >3 classes of antimicrobial drugs, and PDR was defined as characterized by resistance to all antimicrobial drugs, regardless of colistin and polymyxin B susceptibility. XDR was defined as resistance to all antimicrobial drugs. Multilocus sequence typing (MLST) and pulsed-field gel electrophoresis (PFGE) were performed for all PDR isolates according to previously described methods (*5*,*6*). Genes encoding oxacillinases, such as those classified as OXA-23-like, OXA-24/40-like, OXA-51-like, and OXA-58-like, were detected as previously described (*7*). PCR and sequence analyses were performed to detect and characterize the other antimicrobial resistance genes, according to methods reported (*8*).

Of 63 *Acinetobacter* isolates, 31.7% and 34.9% were resistant to imipenem and meropenem, respectively. Of the 63 isolates, 27.0% and 30.2% were resistant to polymyxin B and colistin, respectively. For the other antimicrobial drugs, *Acinetobacter* spp. isolates showed antimicrobial resistance rates >50%. Nineteen isolates (30.2%), all belonging to *A. baumannii*, were PDR. Most of these PDR isolates (16/19, 84.2%) were collected from endotracheal aspirate, and others were from peritoneal fluid and sputum. When characterized by PFGE and MLST, all PDR isolates belonged to a single clone, ST22, and all contained the *bla*_OXA-23_ and *bla*_OXA-66_ genes. IS*Aba1* was detected upstream of *bla*_OXA-23_ and *bla*_OXA-66_ in all PDR isolates. In addition, most PDR isolates contained *bla*_TEM-116_, *bla*_PER-1_, and *bla*_ADC-29_ genes. TEM-116 is a point mutant derivative of TEM-1, Val84→Ile. All β-lactamase genes were located on a plasmid. Also, IS*Aba1* was located at the upstream of all the *bla*_ADC_, which was shown by PCR. However, none of the isolates had *bla*_CTX-M_, *bla*_VEB_, *bla*_IMP_, *bla*_VIM_, or *bla*_GIM_.

Of the PDR isolates, 8 were resistant even to colistin and polymyxin B. These 8 isolates also showed resistance to tigecycline (MICs 4 mg/L). Thus, they were resistant to all antimicrobial drugs tested in this study and were considered to have XDR. The underlying diseases of the patients whose isolates were examined varied (Table). Although 2 isolates with XDR were colonizers, 6 caused infections. All but 1 patient was treated with mechanical ventilation before isolation of the pathogen. Number of hospital days before isolation of *A. baumannii* was 13–256 days, and the number of ICU days before isolation was 2–38 days. Four patients were immunocompromised, and 3 had bacteremia. Among the patients with infections characterized by XDR, the overall 30-day mortality rate was 66.7%, and the infection-related 30-day mortality rate was 50.0%. All 8 isolates with XDR showed common characteristics: ST22 containing OXA-23, OXA-66, TEM-116, PER-1, and ADC-29.

**Table:** Clinical characteristics of 8 patients infected with extremely drug-resistant *Acinetobacter baumannii* isolates, South Korea*

Strain no.	Patient age/sex	Underlying disease	Infection†	Days before isolation		Concurrent bacteremia	30-d outcome	Infection-related death
				Hospitalized	In ICU			
07AC–052	60 y/F	Acute myeloid leukemia	Pneumonia	15	8	No	Died	Yes
07AC–159	79 y/M	Lymphoma	Pneumonia	35	9	No	Died	No
07AC–192	50 y/M	Status postliver transplantation	Pneumonia	256	2	Yes	Survived	NA
07AC–204	55 y/F	Steven-Johnson syndrome	Pneumonia	13	13	Yes	Survived	NA
07AC–336	16 mo/M	Medulloblastoma	Pneumonia	32	13	Yes	Died	Yes
07AC–347	17 mo/M	Hepatoblastoma	Pneumonia	135	28	No	Died	Yes
07AC–329	1 mo/F	Edward syndrome	Colonization‡	33	33	NA	NA	NA
07AC–063	56 y/M	Lung cancer	Colonization‡	26	21	NA	NA	NA

*ICU, intensive care unit; NA, not applicable. All but 1 patient (with strain 07AC–192) had mechanical ventilators. Four patients (with strains 07AC–159, 07AC–192, 07AC–336, and 07AC–063) were immunocompromised hosts who had daily administration of corticosteroid (>20 mg/d of prednisolone or an equivalent drug) during >2 wk and treatment with chemotherapy for an underlying malignancy during 1 month before hospital admission. †Infection is defined as invasion of the body tissues by microorganisms resulting in disease. ‡Colonization occurs when an agent’s presence in a host does not cause a specific immune response or infection.

We report the emergence of XDR in PDR *A. baumannii* isolates in South Korea. Characteristics of PDR *A. baumannii* isolates suggest that they spread from a single clone. A single *A. baumannii* strain with XDR might evolve from the prevailing PDR *A. baumannii* and could disseminate in the ICU, probably after contamination of the hospital environment and by nosocomial transmission. In South Korea, a high resistance rate to imipenem and meropenem in *Acinetobacter* spp. isolates may lead to extensive use of polymyxins (*3*). Thus, we can hypothesize that the most prevalent carbapenem-resistant, or MDR *A. baumannii* clone, became PDR and then evolved into clones with XDR by acquisition of polymyxin resistance caused by antimicrobial pressure. Our investigation showed a simultaneous emergence of resistance to all antimicrobial agents available, including colistin, polymyxin B, and tigecycline. XDR poses serious problems in the treatment of patients with *A. baumannii* infections, especially given the slow development of new antimicrobial agents.
